# Study on an Injectable Chitosan–Lignin/Poloxamer Hydrogel Loaded with Platelet-Rich Plasma for Intrauterine Adhesion Treatment

**DOI:** 10.3390/polym17040474

**Published:** 2025-02-11

**Authors:** Zhipeng Yu, Yang Min, Qi Ouyang, Yuting Fu, Ying Mao, Shuanglin Xiang, Xiang Hu, Liuyun Jiang

**Affiliations:** 1 State Key Laboratory Developmental Biology of Freshwater Fish, School Life Science, Hunan Normal University, Changsha 410081, China15988626517@163.com (Y.F.);; 2 National & Local Joint Engineering Laboratory for New Petro-chemical Materials and Fine Utilization of Resources, College of Chemistry and Chemical Engineering, Hunan Normal University, Changsha 410081, China

**Keywords:** poloxamer, lignin, platelet rich plasma, hydrogel, Intrauterine adhesions

## Abstract

It is a great challenge to obtain an ideal hydrogel for the clinical treatment of intrauterine adhesion (IUA) disease. Here, a novel injectable chitosan–lignin/poloxamer hydrogel loaded with platelet-rich plasma (CL-PF127@PRP) was prepared by self-assembly at room temperature. Fourier transform infrared spectroscopy (FT-IR), scanning electron microscopy (SEM), rheological analysis, and injectable writing were used to characterize the structure of the hydrogel. The results confirmed that the amino group of chitosan and the sulfonic group of sodium lignosulfonate were ionic-crosslinked by electrostatic attraction, which stabilized the three-dimensional structure of the PF127 hydrogel loaded with PRP, and PRP made the porous structure gradually become tight. Moreover, the CL-PF127@PRP hydrogel displayed good injectability and a solid state. The soaking experiment showed that the CL-PF127@PRP hydrogel had suitable degradation at pH = 7 and a good PRP release rate (PRP release 70% at 96 h). Cell experiments in vitro demonstrated that the CL-PF127@PRP hydrogel possessed good biocompatibility, an anti-inflammatory function, and pro-angiogenic activity. Furthermore, an animal experiment of skin wound and IUA confirmed that the skin wound closure rate of the CL-PF127@PRP hydrogel was over 50% on the seventh day. PRP improved the thickness of the endometrium and uterus receptivity, suggesting that the CL-PF127@PRP hydrogel offers great promise for the clinical treatment of IUA.

## 1. Introduction

Intrauterine adhesion is the severe damage of endometrial tissue, leading to partial or total blockage in the uterine cavity. At present, the clinical treatment options are mainly drug treatment or surgical treatment [1,2]. However, these conventional treatment methods have unsatisfactory curative effects or painful treatment processes. Luckily, hydrogels have been widely recognized. For example, some self-healing conductive hydrogels have been widely used in wound healing [3]. Moreover, hydrogels have been used in the study of new treatment schemes for intrauterine adhesions, which can provide a physiological environment similar to the extracellular matrix and is suitable for the physical scaffold inside the uterine cavity [4,5]. However, there is no ideal hydrogel for intrauterine adhesion treatment in the clinical setting. Therefore, it is necessary to explore novel hydrogels for the clinical treatment of intrauterine adhesion.

Polosham 407 (Pluronic F127, PF127) is a triblock copolymer composed of polyoxyethylene and polyoxypropylene units. It is a temperature-sensitive hydrogel with good performance in biomedical medicine, including injectability, good biocompatibility, and other characteristics. It is one of the few polymer materials approved by the FDA, so it is widely used as a carrier for drug delivery, exosomes, or growth factors [6,7]. However, pure PF127 hydrogels have poor adhesion. Sodium lignosulfonate plays a role in controlling drug release [8]. It was reported that modified sodium lignosulfonate hydrogels could adsorb and wrap drugs, and it had a slow-release effect on the drug piperazine ferulate [9,10]. In addition, chitosan is also a natural polymer that has attracted extensive attention in the application of wound dressings, as drug delivery carriers, and as medical hydrogels due to its excellent antibacterial activity [11,12,13]. However, modified chitosan hydrogel is obtained under high temperature, strong acid, long reaction time, and complex operation, which might cause environmental pollution [14]. In addition, the preparation method affects the performance of the hydrogel [15]. It has been reported that chitosan can be ionic-crosslinked using electrostatic interaction [16,17]. Therefore, the sulfonate group in the chemical structure of lignin can form an ionic bond with the amino group of chitosan. Moreover, the complex hydrogel of sodium lignosulfonate and chitosan is expected to further improve the mechanical strength of the PF127 hydrogel.

On the other hand, platelet-rich plasma (PRP) derived from umbilical cord blood contains a variety of biological factors, and it has excellent performance in promoting wound repair and tissue regeneration [18,19,20]. Platelet-derived growth factor (PDGF), transforming growth factor-β (TGF-β), and vascular endothelial growth factor (VEGF) in PRP can promote cell proliferation, differentiation, and angiogenesis [21,22]. Hydrogel-loaded PRP accelerated wound healing by promoting angiogenesis and nerve repair of diabetic wounds [23]. Moreover, PRP has been applied to endometrial research in recent years, such as repeated implantation failure (RIF), chronic endometritis (CE), and IUA [24,25,26]. It was shown that intrauterine injection of PRP could restore endometrial thickness and restore pregnancy with good therapeutic effect [27]. Jang et al. found that PRP could not only stimulate and accelerate the regeneration of the endometrium but also reduce the damaged endometrium fibrosis degree in a rat model [28]. Kim et al. reported that the fibrosis was reduced after PRP injection in mice with uterine damage, and PRP promoted endometrial regeneration and improved pregnancy outcomes [29]. However, whether PRP can be loaded in the chitosan–lignosulfonate/PF127/PRP (CL-PF127/PRP) hydrogel and exert the endometrial regeneration promotion effect has not been reported.

Based on these, in the present study, we attempted to prepare a novel injectable hydrogel of CL-PF127@PRP by self-assembly at room temperature. The formation, rheology, injectability, and microstructure of the hydrogel were studied by IR analysis, SEM observation, rheology tests, and injectable writing. Moreover, degradation and drug release of the complex hydrogel were investigated by soaking for different periods of time. In addition, the biocompatibility of the complex hydrogel was evaluated by co-culturing with HUVEC and HESC cells, and MTT and AO fluorescence staining were conducted. Anti-inflammation and angiogenesis were checked by RT-qPCR and Western blot. Furthermore, skin wound recovery and regeneration were explored in ICR mice, and intrauterine adhesion in SD rats was analyzed by gross observation, average thickness of dermal cortex, HE and Masson staining, and immunohistochemistry for angiogenesis exploration. The main purpose was to explore the feasibility of the CL-PF127@PRP hydrogel for intrauterine adhesion treatment.

## 2. Materials and Methods

### 2.1. Materials and Platelet-Rich Plasma (PRP) Preparations

Poloxamer (PF127) with a molecular weight of 1000~7000 was purchased from Sigma. Chitosan (CS) was bought from Shanghai Biotechnology Co., Ltd. (Shanghai, China) with a deacetylation degree of 80% and a molecular weight of 2 × 10^5^. Sodium lignosulfonate was brought from Shanghai Aladdin Biochemical Technology Co. Ltd. (Shanghai, China).

The pre-collected umbilical cord blood (10 mL) was centrifuged, and the upper two layers were collected and centrifuged again. After discarding about 75% of the clear light yellow liquid in the upper layer, it was gently mixed and re-suspended as platelet-rich plasma (PRP). Appropriate amounts of PRP and whole blood were used for blood cell analysis; when the platelet content reached more than 5 times that of the whole blood, the PRP preparation was confirmed to be successful. Then, 50 U/mL thrombin calcium chloride solution was added to the PRP in a ratio of 1:9 and incubated at 37 °C for 1 h for activation. The activated PRP supernatant was placed at −80 °C on standby [30].

### 2.2. Preparation of the CL-PF127@PRP Hydrogel

Chitosan powder (0.15 g) was dissolved in a 2% acetic acid solution to prepare a 3 wt% chitosan solution, and 0.1 wt% lignin aqueous solution with 0.15 g powder was added with magnetic stirring for 6 h until evenly mixed. Then, the solution was added into a 35 wt% PF127 aqueous solution containing 5.38 g poloxamer powder. Next, PRP and water with a volume ratio of 1:5 were introduced, and the solution was shaken overnight at 4 °C using a shaker. The CL-PF127@PRP hydrogel was finally obtained. In addition, CL-PF127 and 35 wt% PF127 hydrogels were prepared according to a similar process as the controls.

### 2.3. Structural Characterization

Fourier transformation infrared (FTIR) analysis of the chitosan, lignin, poloxamer, and CL-PF127@PRP hydrogels was carried out by a Thermo Niclet 670 spectrometer in the absorbance mode at a wavelength range between 500 and 4000 cm^−1^.

The hydrogel was treated by freeze-drying. After spraying gold, the hydrogel was observed by a scanning electron microscope (SEM).

The rheological property was tested, and the storage modulus (G′) and loss modulus (G″) in the range of 5–40 °C were measured in the continuous scanning mode with constant stress of 0.5 Pa and fixed frequency of 1 Hz. Moreover, the change curves of storage modulus (G′) and loss modulus (G″) with time under different shear strains were obtained for 5 time periods with 0.1% and 100% strain alternating scanning, showing a linear distribution. The frequency was set to a constant value of 1 Hz for testing to observe the recovery of the hydrogel under high strain damage.

The injectable property of the hydrogel was validated by injectable writing. The hydrogel solution was squeezed out with a 10 mL disposable medical syringe without a needle, whose inner diameter was 1.2 mm, and the English letters of “HNNU” were written.

### 2.4. In Vitro Soaking of Hydrogels

CL-PF127@PRP samples with a strip size of 50 × 5 × 0.1 mm^3^ were placed into a sealed propylene tube with 10 mL phosphoric acid buffer solution (PBS) at 37 °C. The samples were removed after being soaked for 1, 2, 3, 4, 5, 6, and 7 days and washed with deionized water. The surface water was absorbed with filter paper. The original weight and dry weight of the sample were noted as *W*_1_ and *W*_2_, respectively. The weight loss ratios were calculated as follows [31]:

$$\mathrm{Weight}\;\mathrm{loss}/\%=\frac{W_{2}-W_{1}}{W_{1}}\times 100\%$$

### 2.5. In Vitro PRP Release of CL-PF127@PRP Hydrogels

About 0.2 g samples were placed into 100 mL of PBS solution with pH 6, 7, and 8 and stirred evenly at room temperature. Then, 2 mL solutions were taken out at corresponding time points of 4 h, 12 h, 24 h, 48 h, 60 h, 72 h, and 96 h in turn, and 2 mL PBS was added into the solution. The PRP concentration of the composite hydrogel was measured with an ultraviolet–visible spectrophotometer (UV-2450, Tokyo, Japan), and the PRP release curve was drawn [32].

### 2.6. Preparation of the Hydrogel Extract

The hydrogels with or without PRP were taken out at 4 °C and grouped into three duplicate groups. Then, 1 mL from each group was taken and added into a 2 mL centrifuge tube, which was placed in a 37 °C incubator for solidification. Next, 500 µL of the corresponding cell culture medium was added for incubation at 37 °C for 48 h. The supernatant was prepared by filtering and sterilization.

### 2.7. Cell Culture

The human umbilical vein endothelial cell line (HUVEC) was obtained from ATCC and frozen in our laboratory. Human endometrium mesangial cells (HESCs) were a gift from Nanchang University School of Medicine. After thawing, 3 mL of preheated culture medium was added to the centrifuge tube, and the melted cell solution was quickly added and centrifuged at 800 rpm for 3 min. The supernatant was discarded and replaced with 1 mL of new culture medium to re-suspend the cell pellet and then cultured. The medium was changed every 6–8 h. When the cells were fully grown, passage procession was performed. When the cells did not adhere to the wall, culture medium was added to terminate digestion, gently blowing the bottom of the culture dish and down all the cells. The cell suspension was added to a new culture dish containing the new culture medium according to the experimental requirements. It was gently cross-shaken and mixed well.

### 2.8. Biocompatibility

Cells of HUVEC and HESCs were added with a cell density of 8000 cells/well and cultured for 12 h. Extraction solution was added, and the culture medium was sucked off. Then, 100 μL of 5 mg/mL MTT was added and co-cultured at 37 °C for 4 h. The liquid was removed, and 200 μL of DMSO was added to each well in the dark and shaken for 10 min. Finally, the absorbance of the well plate was detected using an enzyme-linked immunosorbent assay (ELISA) reader at a wavelength of 490 nm [33].

Moreover, the sample was fixed with 95% ethanol for 10 min, and 1% acridine orange staining solution was used to dye for 1 min. The sample was washed with PBS 5 times after dyeing, the excess dye was removed, and PBS was added. The sample was then observed under a fluorescence microscope.

### 2.9. RT-qPCR Testing

RNA was extracted with Trizol reagent. Then, 4 xg DNA wiper mix and 1 µg template were added according to the RNA instructions. Sterile water was added to make a total reaction volume of 16 µL at 42 °C for 2 min after mixing to remove genomic DNA. Then, 4 µL of 5× mix was added into the EP tube in the previous step, and the total volume was 20 µL. The reverse transcription reaction was carried out after mixing at 50 °C for 15 min and 85 °C for 5 s. Finally, the reverse transcripts were diluted 10 times with sterile water, and the mixed reaction solution was prepared in an EP tube with 5 µL of 2× sybr mix, 0.2 µL of upstream and downstream primers, 1 µL of cDNA, and 3.6 µL of sterile water for a total volume of 10 µL; each sample was repeated three times. After the sampling was completed, we went on the machine and started detection according to the procedure of RT-qPCR [34].

### 2.10. Western Blot

After cellular protein was extracted, separate gel and concentrated gel were prepared according to the SDS-PAGE dispensing table, which was pre-electrified for 40 min at a voltage of 120 V and a current of 10 mA. When bromophenol blue ran to condensation at the bottom, the electrophoresis was completed. The pre-cooled film transfer buffer was poured into the special cylinder to transfer film, and the black-and-white film transfer clamp was placed into the cylinder. The gel was carefully taken out and placed into the rotating film clamp after electrophoresis. The cut PVDF membrane was taken out and placed into methanol for activation for 10 s, which was also placed into the rotating membrane clamp with the condition of 100 V, 400 mA, and 90 min. After rotating the membrane, the membrane was placed into 5% skim milk and sealed. It was shaken for 2 h, and the sealing was completed. The sealing liquid was discarded, and the PVDF membrane was cleaned three times with 1XTBST vibration for 10 min each time. The PVDF membrane was cut according to the instructions of the marker, and the corresponding strip anti-liquid was added and shaken overnight at 4 °C. After recovering the primary reactance, the membrane was washed with 1XTBST three times for 10 min each time. The corresponding secondary antibody was incubated according to the source of the primary antibody and in a shaking table at room temperature for 1 h. The secondary antibody was recovered, and the membrane was washed with 1XTBST three times for 10 min each time. The developer with a 1:1 ratio was used for development [35].

### 2.11. Construction of a Cellular Inflammation Model

The digestive cells were seeded in a six-well plate. After the cell density reached about 80%, the treatment group was replaced by 40 µM mifepristone medium with a low serum concentration, and the control group was added with anhydrous ethanol, which dissolved the drug in the same concentration volume. After 48 h, RT-qPCR was used to detect the expression of related inflammatory factors and the NF-kB signaling pathway as well as the expression of the marker inflammatory factors of 40 μM mifepristone (TNF-α, IL-1β, and IL-6). The relative mRNA of the NF-kB signaling pathway-related proteins was used to confirm the successful construction of the inflammation model. The mRNA level of each group was detected by RT-qPCR, and the protein level of the NF-kB pathway was detected by Western blot [36].

### 2.12. Angiogenesis Experiment

The subpackaged matrix adhesive was thawed at 4 °C and diluted tenfold with the medium. Then, 50 µL of diluted matrix adhesive was added into a 96-well pre-cooled plate, which was placed in the cell incubator and allowed to solidify. The HUVEC cells were digested and counted with a cell density of 2 × 10^4^ cells/well. After the cells adhered to the wall, different extraction mediums were changed, and tube formation was observed after about 4 h. To further confirm the pro-angiogenic effect of PRP, the levels of angiogenesis-related proteins of VEGFR1 and VEGFR2 were detected by Western blot [37].

### 2.13. Animal Experiment

Sixteen male ICR mice (4 weeks old) and sixteen female SD rats (7 weeks old) were purchased from Changsha Tianqin Biotechnology Co., Ltd. (Changsha, China) They were fed for one week before the animal experiment. For the skin wound modeling, the mice were anesthetized with 1% pentobarbital sodium. The back hair was trimmed with a hair clipper, and a circular wound was created on the back with an 8 mm skin puncher. The mice were randomly divided into four groups, and 20 µL of medication was administered immediately for wound modeling according to each group. Observation and photography of wounds were conducted on the same day as well as 3 days and 7 days after wound modeling [38].

For IUA modeling, the rat was anesthetized with 2% pentobarbital sodium and placed on the operating table. The abdominal hair was cut off, and the urethral opening of about 2 cm above was opened. The intersection of the Y-shaped uterus below the bladder and the uterine horn along the uterine canal was cut and opened at the uterine horn. Then, 1 mL of 95% alcohol was injected for 3 min. The pre-prepared disposable sterile 50 mL syringe was inserted into the uterine tube, and the entire uterine tube was gently rubbed repeatedly for 10 min. After the injury was completed, the uterine gap was sutured with absorbable sutures, and the anatomical position of the uterus was restored and sutured for the abdomen layer by layer; thus, the intrauterine adhesion model was made. The rats were euthanized after two weeks, and uterine tissue was sampled and quickly placed in a 4% paraformaldehyde fixative [39].

After the experiment, Image J software was used to measure the wound area in the photos and calculate the relative wound area ratio based on the size of the wound area. The calculation formula was the relative wound area ratio (%) = daily wound area/original wound area × 100%. The average thickness of dermal cortex was measured and quantitatively statistically analyzed. HE and Masson staining were conducted, and the expression of CD31 was detected by immunohistochemistry for angiogenesis exploration.

### 2.14. Statistical Analysis

All data were analyzed using GraphPad Prism 9.0 and Image J software, and mean ± standard deviation was calculated. Using *t*-test analysis to compare the differences between two groups, *p* < 0.05 was considered to be a statistically significant difference.

## 3. Results and Discussion

### 3.1. Characterization of the CL-PF127@PRP Hydrogel

#### 3.1.1. FT-IR Analysis

**Figure 1:**
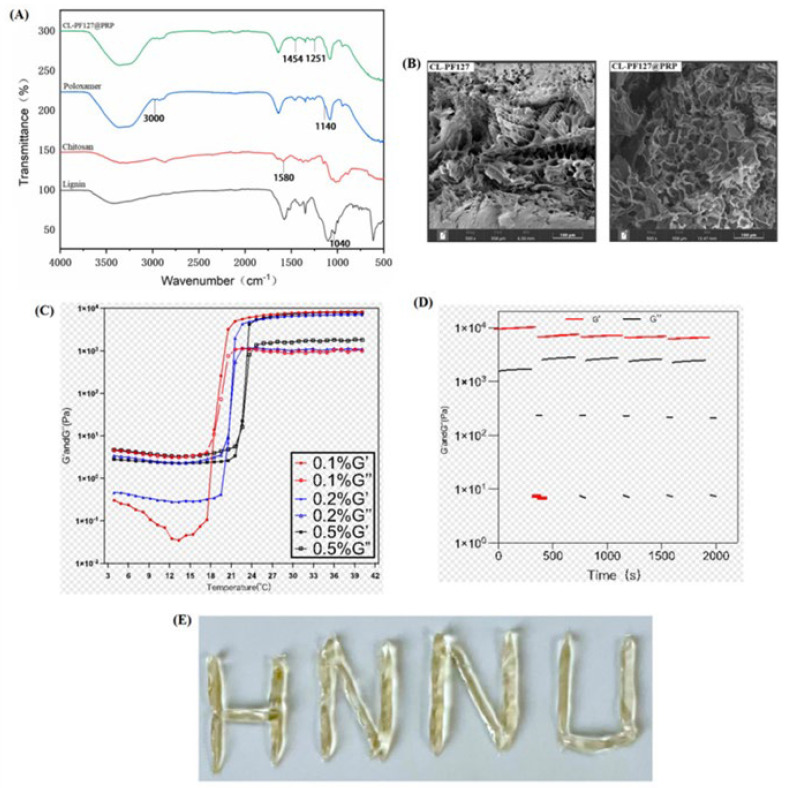
(**A**) FT-IR spectrum of samples. (**B**) SEM micrographs of two hydrogels. (**C**) The relationship between the rheological properties of the CL-PF127@PRP hydrogel and temperature. (**D**) Rheological performance test with 5 cycles of 1% and 100% stress for 300 s and 60 s, respectively. (**E**) Alphabet for hydrogel injection.

Figure 1A shows the characteristic peaks of lignin, chitosan, poloxamer, and the CL-PF127@PRP hydrogel. The C-H stretching vibration peak of poloxamer appeared at about 3000 cm^−1^, while the C-C skelet stretching vibration peak appeared at about 1140 cm^−1^. The bending vibration peak of chitosan amino was about 1580 cm^−1^, and the characteristic peak of 1040 cm^−1^ belonged to the sulfonic acid group in sodium lignosulfonate [40]. A new characteristic peak was present in the composite hydrogel, suggesting that the ionic crosslinking was generated between the amino group of chitosan and the sulfonic group of sodium lignosulfonate by electrostatic attraction, which further stabilized the three-dimensional structure of the hydrogel.

#### 3.1.2. SEM Observation

Figure 1B gives the morphologies of the composite hydrogel with and without PRP. As can be seen, the three-dimensional structure of loose pores was clearly visible. With the addition of PRP, the number of pores decreased, and the reticular structure gradually became tight, suggesting that the CL-PF127@PRP hydrogel had good structural stability.

#### 3.1.3. Rheological Analysis

Rheological experiments reflected the properties of the hydrogel. The addition of chitosan and lignin did not affect the temperature sensitivity of poloxamer. When 0.1 wt% lignin was added, the hydrogel exhibited a slightly higher storage modulus (Figure 1C). Moreover, 1% and 100% strain were selected to scan alternately in the time period, and the CL-PF127@PRP hydrogel had a complete internal structure at 1%, while the internal structure was changed at 100% strain. However, the storage modulus (G′) and loss modulus (G″) of the hydrogel could be reduced in multiple alternating cycles, and it could recover quickly and had the ability of rapid network recovery (Figure 1D), which would greatly reduce the risk of being washed away and provide better adherence to the wound in a wet environment. Moreover, the transformation of solid–liquid phase directly confirmed the injectability of the hydrogel.

#### 3.1.4. Injectability Test

The syringe was used to inject writing letters. The hydrogel had good injectability and solid state, indicating the CL-PF127@PRP hydrogel possessed good structural stability (Figure 1E), which would be essential for intrauterine adhesion treatment in vivo application.

### 3.2. In Vitro Soaking of the Poloxamer–Chitosan/Lignin/PRP Hydrogel

#### 3.2.1. Weight Loss of Samples After Degradation

**Figure 2:**
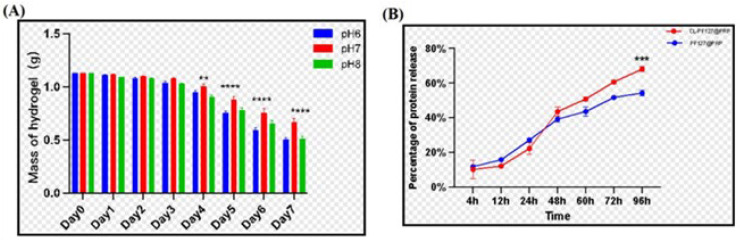
(**A**) Degradation rate of the CL-PF127@PRP hydrogel with time at different pH values. (**B**) The total protein release rate of the PF127@PRP and CL-PF127@PRP hydrogels (*p* < 0.05 (*), <0.01 (**), <0.001 (***), <0.0001 (****)).

Figure 2A shows the weight loss of the composite hydrogel immersed in PBS with different pH values. The mass of hydrogel decreased over time, showing that the hydrogels were degradable, which was attributed to the natural biodegradation of chitosan and lignin. However, the hydrogels had different weight loss rates at different pH values, and pH = 6 or pH = 8 exhibited more weight loss, while pH = 7 was the most moderate degradation condition, which would be conducive to gradually releasing PRP to maximize the therapeutic effect.

#### 3.2.2. Release of PRP of the Poloxamer–Chitosan/Lignin/PRP Hydrogel

The release of PRP in the hydrogel is given in Figure 2B. As can be seen, PRP in the hydrogel slowly released within 96 h; there was no sudden release phenomenon. Moreover, it was noted that PRPP was released more slowly from the hydrogel containing lignin and chitosan than from the single pure poloxame hydrogel before 48 h; however, after 48 h, PRP was released a little faster, which might be attributed to the faster degradation. The greater release amount might be more advantageous for tissue repair.

### 3.3. Biocompatibility of Hydrogels

#### 3.3.1. MTT Test

**Figure 3:**
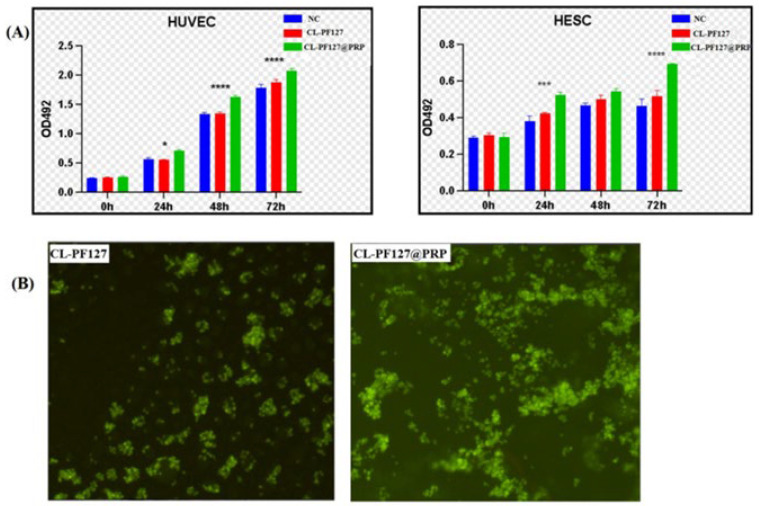
Biocompatibility of the CL-PF127@PRP hydrogel extract. (**A**) The cell proliferation of HUVECs and HESCs. (**B**) AO fluorescence staining photos of hydrogel after co-culture for 24 h (*p* < 0.05 (*), <0.01 (**), <0.001 (***), <0.0001 (****)).

The biocompatibility of the hydrogel is the primary factor for its application. A cytotoxicity test was performed by MTT assay, which is usually used to detect cell viability, and the extract medium was co-cultured with HUVEC and HESC for 72 h. The OD value versus time is given in Figure 3A. It can be seen that the OD value increased with the extension of culture time, showing that the extract of hydrogel had no negative impact on the proliferation of two kinds of cells compared with the control group. Moreover, the addition of PRP led to higher OD values, suggesting that PRP could promote cell proliferation.

#### 3.3.2. AO Fluorescence Staining

To further explore the effect of the hydrogel on cell viability, the cells were fixed and stained by AO fluorescence staining after 24 h, and the cells were observed under the light microscope. It could still be seen that most of the living cells were stained with green fluorescence (Figure 3B), indicating that the hydrogel was basically non-toxic to cells, which explained the biocompatibility of the hydrogel. Moreover, it could be seen that the number of cells on the CL-PF127@PRP hydrogel was more than on the CL-PF127 hydrogel, which further demonstrated that PRP had good biocompatibility.

### 3.4. Anti-Inflammatory

**Figure 4:**
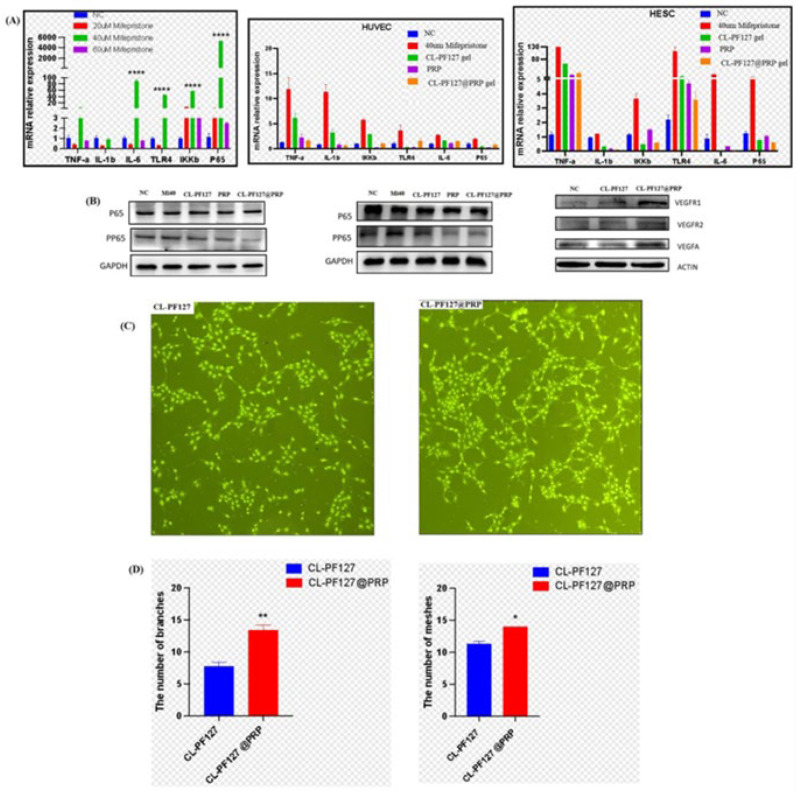
The hydrogel inhibits inflammation and promotes angiogenesis in vitro. (**A**) Mifepristone concentrations of 20 µm, 40 µm, and 60 µm were used to treat cells to establish a cellular inflammation model and RT-qPCR. The expressions of pro-inflammatory factors and NF-kB in HUVECs and HESCs were detected in each treatment group. (**B**) Western blotting of the expression of NF-kB-related proteins in HUVECs and HESCs in the treatment groups and the expression of angiogenic proteins in HUVECs treated with the CL-PF127 hydrogel and the CL-PF127@PRP hydrogel extract. (**C**) AO fluorescence staining of the angiogenic map of HUVECs treated with the CL-PF127 hydrogel and the CL-PF127@PRP hydrogel extract. (**D**) Quantitative analysis of angiogenesis branches and meshes of the CL-PF127 hydrogel and the CL-PF127@PRP hydrogel. “*” is NC and mifepristone groups; “#” is mifepristone and treatment groups (*p* < 0.05 (*/#), <0.01 (**/##), <0.001 (***/###), <0.0001 (****/####).

After confirming the good biocompatibility of the hydrogel, the medicine mifepristone was used to treat cells to establish a cell inflammatory injury model. To determine the appropriate drug treatment concentration, mifepristone concentrations of 20 µm, 40 µm, and 60 µm were selected to treat the cells. After 48 h, RT-qPCR was used to detect the expression of related inflammatory factors and the NF-kB signaling pathway as well as the expression levels of the marker inflammatory factors of 40 µm mifepristone (TNF-ɑ, IL-1β, and IL-6). The relative mRNA of the NF-kB signaling pathway-related proteins ws the most stable and significant, indicating that the inflammatory injury model was successfully established (Figure 4A). Mifepristone of 40 µm concentration was used for subsequent experiments. After the cells of HUVEC and HESC were treated with mifepristone for 48 h, the hydrogel extract of each group was used for salvage treatment, and the mRNA levels of each group were detected by RT-qPCR. The results indicated that the CL-PF127@PRP hydrogel extract could successfully reduce the levels of inflammatory factors, NF-kB, and its upstream mRNA. PRP and hydrogel extract could effectively alleviate cell inflammation, especially the hydrogel extract containing PRP (Figure 4B). Moreover, Western blot was used to detect the protein levels related to the NF-kB pathway. The results showed that the level of nuclear phosphorylated p65 (p-p65) protein decreased after treatment with the CL-PF127@PRP hydrogel extract, suggesting that it alleviated inflammation by inhibiting the NF-kB signaling pathway.

### 3.5. Angiogenesis Promotion

The hydrogel extract was co-cultured with HUVEC. It could be seen that the number of tubes and grids in the hydrogel with the PRP extract group was more than that in the hydrogel without the PRP extract group (Figure 4C). Quantitative analysis results also showed that the CL-PF127@PRP group had more branches and grids than the CL-PF127 group. To further confirm the pro-angiogenic effect of PRP, the levels of angiogenesis-related proteins were detected by Western blot. As expected, in the CL-PF127@PRP hydrogel extract group, the protein levels of vascular endothelial growth factor A (VEGFA) and two vascular endothelial growth factor receptors (VEGFR1 and VEGFR2) were up-regulated (Figure 4D). Therefore, the CL-PF127@PRP hydrogel could promote cell angiogenesis.

### 3.6. Animal Experiment

#### 3.6.1. Skin Injury Repair in Mice

**Figure 5:**
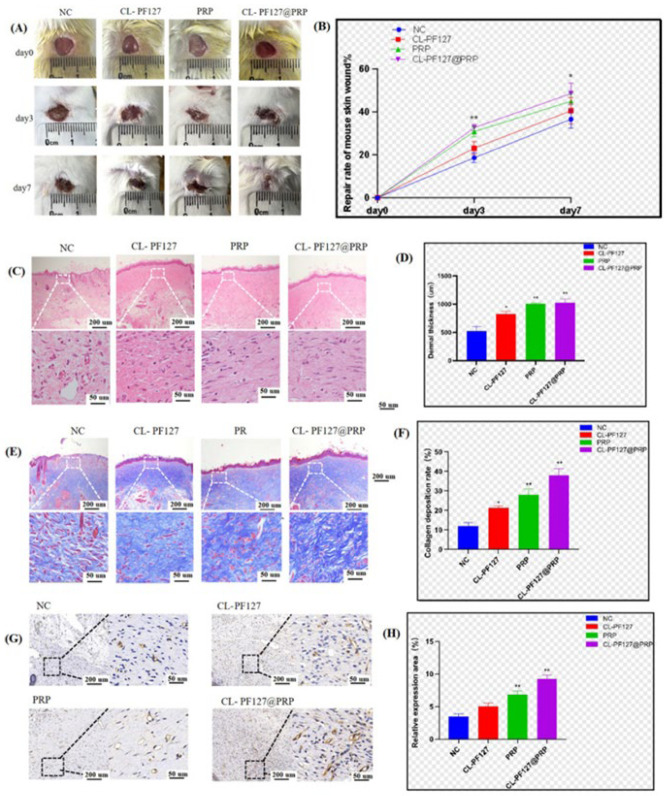
Skin damage repair in mice. (**A**) Recovery diagram of mouse skin wound. (**B**) Statistical chart of skin wound closure rate in mice. (**C**) HE staining of wound tissue incision. (**D**) Statistical chart of dermis thickness of the wound section. (**E**) Masson staining of the wound tissue incision. (**F**) Statistical chart of the collagen deposition rate in the wound section. (**G**) CD31 immunohistochemistry of the wound tissue incision. (**H**) Statistical chart of the relative positive cell number and expression (*p* < 0.05 (*), <0.01 (**), <0.001 (***), <0.0001 (****)).

The mice were randomly divided into four groups and treated with physiological saline, CL-PF127, PRP, and CL-PF127@PRP. The closure of the wound was recorded with a camera on days 0, 3, and 7 (Figure 5A). The photos of wounds and the statistical chart of the wound healing rate of each group were analyzed (Figure 5B). On the third day after the establishment of the wound model, the wounds of animals of each group had different closure degrees. The wound closure in the PRP and CL-PF127@PRP groups was more ideal, but there was no significant difference between the two groups. On the seventh day, the wound closure area of the CL-PF127@PRP hydrogel group was larger than that of the CL-PF127 group, indicating that PRP could promote skin tissue regeneration and would be beneficial for the recovery of wounds. The sustained-release performance of the hydrogel maximized the therapeutic effect of PRP.

To further explore the effect of the CL-PF127@PRP hydrogel on wound tissue recovery, Figure 5C gives HE staining photos of the recovery status of fibroblasts in the epidermal and dermal layers of the wound. It was found that the regeneration status of the epidermal layer in the NC group was the worst; there was incomplete formation of epidermis and sparse arrangement of fibroblasts in the dermis layer. Meanwhile, the CL-PF127@PRP hydrogel group had a more completely recovered epidermal layer, and the dermal fibroblasts were arranged regularly and closely, so the recovery was closest to normal skin tissue. In addition, quantitative statistical analysis of the average thickness of the dermis layer revealed that the thickness of the dermis layer in each treatment group was significantly higher than that in the NC group, and the thickness of the dermis in the CL-PF127@PRP hydrogel group was the highest (Figure 5D).

Figure 5E shows the Masson staining photos. As we know, the principle of Masson staining is that collagen fibers have high permeability to dyes, resulting in a blue color for collagen fibers and a red color for muscle fibers due to their dense structure that makes it difficult for dyes to penetrate. Therefore, the collagen amount can be evaluated by the produced blue depth. During the process of wound tissue recovery, the recovery of the dermis layer plays a dominant role. The dermis layer is mainly composed of fibroblasts, so the collagen fibers are an important indicator of fibroblast composition. It could be seen that the CL-PF127@PRP hydrogel group displayed the darkest blue color, indicating that the collagen deposition in this group was the most, and the wound tissue recovered closest to the normal skin tissue. However, the NC group had the lightest blue color, showing it had the lowest collagen deposition density and a lower tissue regeneration. Figure 5F shows the quantified statistical analysis for collagen deposition content. The analysis results indicated that the CL-PF127@PRP hydrogel group possessed the largest collagen deposition, and the PRP group was higher than that of CL-PF12 and the NC group, which was consistent with the visual observation. PRP could promote collagen fiber deposition, thereby accelerating the dermis layer regeneration and facilitating wound recovery, so the CL-PF127@PRP hydrogel effectively enhanced the therapeutic effect.

Angiogenesis is crucial for the regeneration of the dermis layer. In the early stage of wound healing, a large amount of neovascularization would bring a lot of oxygen and nutrition to the wound tissue belt to promote wound healing. The number of newly formed blood vessels can be used as one of the signs of wound recovery. The platelet endothelial cell adhesion molecule CD31 is highly expressed in vascular endothelial cells, and the marker is commonly used to evaluate angiogenesis. The expression of CD31 was detected by immunohistochemistry, and a large number of brown particles were observed in the CL-PF127@PRP hydrogel group (Figure 5G), indicating it had high expression of positive cells, followed by the PRP group and the CL-PF127 group. There was a significant difference in the number of positive cells between the CL-PF127@PRP hydrogel group and the NC group (Figure 5H), proving that PRP could stimulate angiogenesis and promote the recovery of wound tissue. A large amount of growth factors were derived from platelet particles after the activation of PRP from the CL-PF127@PRP hydrogel, promoting angiogenesis.

According to the results of HE and Masson staining and immunohistochemistry, it was confirmed that PRP could significantly promote the proliferation of dermal and epidermal cells and produce neovascularization formation; thus, the CL-PF127@PRP hydrogel could effectively accelerate skin wound healing in a mice model.

#### 3.6.2. Intrauterine Adhesion Repair in SD Rats

To continue investigating the ability of the CL-PF127@PRP hydrogel to repair and regenerate tissue in the body, an intrauterine adhesion model in a rat was constructed. The rats were randomly divided into four groups and treated with CL-PF127, PRP, and CL-PF127@PRP, with physiological saline acting as the control group. After two weeks, the rats were sacrificed, and the uterine tissue was sampled for histological examination to evaluate the treatment effect of the CL-PF127@PRP hydrogel on uterine cavity tissue.

**Figure 6:**
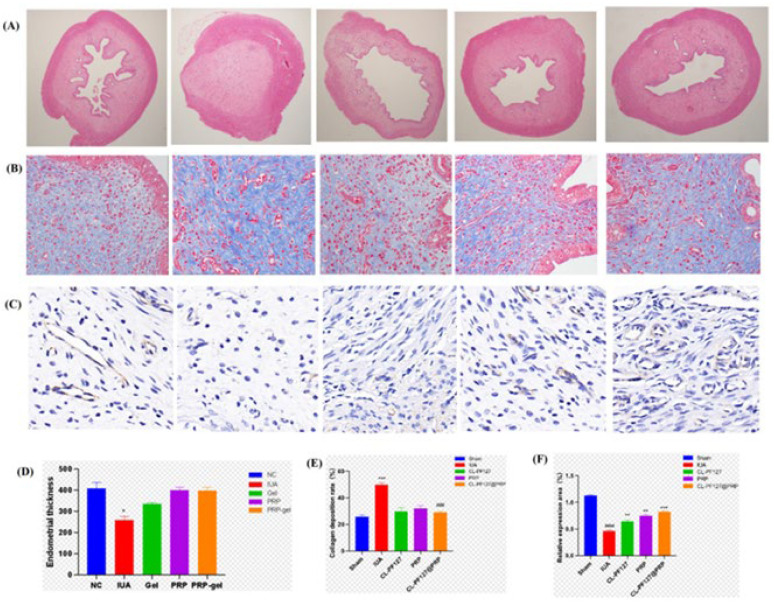
Intrauterine adhesion repair in rats. (**A**) HE staining of uterine tissue sections. (**B**) Masson staining of uterine tissue sections. (**C**) Immunohistochemistry of CD31 in uterine tissue sections. (**D**) Statistical chart of endometrial thickness. (**E**) Statistical chart of the collagen deposition rate in the uterine cavity. (**F**) Statistical chart of relative positive cell number and expression. “*” is NC and mifepristone groups; “#” is mifepristone and treatment groups (*p* < 0.05 (*/#), <0.01 (**/##), <0.001 (***/###), <0.0001 (****/####).

According to the HE staining results, there were almost no gaps in the uterine cavity, and the endometrial structure was completely destroyed. The myometrium was necrotic in a large area compared with the sham operation group, and even thrombosis could be seen in the model group, which indicated that the IUA damage model was successfully established. After treatment by the CL-PF127@PRP hydrogel, the morphology of the uterine cavity was almost close to that in the normal sham operation group, and the regeneration of endometrial epithelial cells was good (Figure 6A). Quantitative statistical analysis also showed that the endometrial thickness of each treatment group recovered to varying degrees, which was different from that of the sham operation group, but there was no significant difference (Figure 6D).

Masson staining was used to evaluate and analyze endometrial fibrosis. The blue part represents collagen fibers. Endometrial fibrosis is one of the main pathological features of IUA and can be used to evaluate the pathological changes of uterine tissue, while the fibrosis degree can be used as an important indicator of IUA. Compared with the sham operation group, the model group contained a large amount of blue collagen fibers, showing it had severe intrauterine fibrosis. Each treatment reduced the blue collagen fiber deposition, meaning the uterine cavity fiber status was improved (Figure 6B). Quantitative analysis of the collagen deposition ratio is given in Figure 6E. It can be seen that the collagen deposition ratio in the model group was 49.68%, which was significantly higher than that in the sham operation group (25.6033%). Among the treatment groups, collagen deposition ratios of the CL-PF127 group, PRP group, and CL-PF127@PRP group were 29.82%, 31.99%, and 28.9677%, respectively. Obviously, the collagen fibrosis deposition ratio in each group was significantly reduced compared with the IUA model group. CL-PF127@PRP especially had the best inhibition effect on collagen fibrosis. Moreover, the collagen deposition ratios were lower than those of self-healing P10G20 hydrogels reported in the relative literature [5]. The endometrial fibrous tissue area was 50%, suggesting CL-PF127@PRP possesses better application prospects for IUA prevention.

Neovascularization in endometrial tissue is one of the indicators to evaluate endometrial tissue regeneration. Immunohistochemistry was performed on the endometrial tissue sections of rats. The angiogenesis marker CD31 was detected (Figure 6C), and the proportion of positive cells in the slice tissue was observed (Figure 6F). Compared with the sham operation group, the number of positive cells decreased significantly. In the CL-PF127@PRP hydrogel group, the expression level of CD31 and the number of positive cells were restored and significantly increased, indicating the CL-PF127@PRP hydrogel could effectively promote angiogenesis.

According to the results of the intrauterine structure and the endometrial thickness of uterine tissue in rats by HE staining, the intrauterine fibrosis in rats by Masson staining, and the number of neovascularization by the immunohistochemical section of CD31, it was inferred that the main mechanism of the CL-PF127@PRP hydrogel in the treatment of IUA was the hydrogel gelling in the body, which formed a physical barrier and played a slow-release role in carrying PRP substances. CL-PF127@PRP had good biocompatibility due to the chitosan, lignin, and PF127, so it could provide a platform for endometrial cell proliferation and stimulate neovascularization so as to improve the thickness of the endometrium and increase the receptivity of the uterus.

## 4. Conclusions

To sum up, in this work, a novel injectable CL-PF127 hydrogel loaded with PRP was prepared. FT-IR analysis confirmed that the amino group of chitosan and the sulfonic group of sodium lignosulfonate were ionic-crosslinked by electrostatic attraction, which stabilized the three-dimensional structure of the PF127 hydrogel loaded with PRP. Chitosan and lignin did not affect the temperature sensitivity of the PF127 hydrogel, and PRP made the porous structure gradually become tight. Moreover, the CL-PF127@PRP hydrogel displayed good injectability and a solid state. The soaking experiment showed that the CL-PF127@PRP hydrogel had suitable degradation and PRP release rates. Cell culture experiments in vitro demonstrated that the hydrogel possessed good anti-inflammatory function and pro-angiogenic activity, which was also well confirmed by animal experiments on a mouse skin wound model and an SD rat intrauterine adhesion model in vivo, suggesting that the CL-PF127@PRP hydrogel has excellent biological function. This study provides a new CL-PF127@PRP hydrogel for the clinical treatment of intrauterine adhesion. Meanwhile, it can open a new biomedical application pathway for natural polymers of chitosan and lignin.

## Data Availability

The original contributions presented in this study are included in the article. Further inquiries can be directed to the corresponding authors.
